# Applying the RE-AIM framework in a process evaluation of the introduction of the Non-Pneumatic Anti-Shock Garment in a rural district of Zimbabwe

**DOI:** 10.1371/journal.pone.0251908

**Published:** 2021-05-20

**Authors:** Thulani Leslie Magwali, Abaden Svisva, Tarryn Bowers, Fishiwe Chiyaka, Jenna-Dawn Conway, Bernard Madzima, Violet Mambo, Alexio Mangwiro, Andy Elizabeth Carmone

**Affiliations:** 1 Department of Obstetrics and Gynaecology, University of Zimbabwe, Harare, Zimbabwe; 2 Clinton Health Access Initiative, Harare, Zimbabwe; 3 Maternal Health Program, Zimbabwe Ministry of Health, Harare, Zimbabwe; LSHTM, UNITED KINGDOM

## Abstract

The Non-Pneumatic Anti-Shock Garment (NASG) is a first aid tool that can halt and reverse hypovolemic shock secondary to obstetric hemorrhage. The World Health Organization recommended the NASG for use as a temporizing measure in 2012, but uptake of the recommendation has been slow, partially because operational experience is limited. The study is a process evaluation of the introduction of NASG in a public sector health facility network in rural Zimbabwe utilizing an adapted RE-AIM, categorizing observations into the domains of: reach, effectiveness, adoption, implementation and maintenance. The location of the study was Hurungwe district, where staff members of 34 health facilities at primary (31), secondary (2) and tertiary (1) levels of care participated. We found that all facilities became skilled in using the NASG, and that the NASG was used in 10 of 11 instances of severe hemorrhage. In the cases of hypovolemic shock where the NASG was used, there were no maternal deaths and no extreme adverse outcomes related to obstetric hemorrhage in the study period. Among the 10 NASG uses, the garment was used correctly in each case. Fidelity to processes was high, especially in regard to training and cascading skills, but revisions of the NASG rotation and replacement operating procedures were required to keep clean garments stocked. Clinical documentation was also a key challenge. NASG introduction dovetailed very well with pre-existing systems for obstetric emergency response, and improved clinical outcomes. Scale-up of the NASG in the Zimbabwean public health system can be undertaken with careful attention to mentorship, drills, documentation and logistics.

## Introduction

Preventable maternal death remains a seemingly intractable problem globally, despite availability of a wide spectrum of interventions to address the key drivers in maternal mortality, including obstetric hemorrhage (OH), one of the most common causes of maternal mortality in the world [1]. In 2019, the Multiple Indicator Cluster Survey reported maternal mortality in Zimbabwe as 462 per 100,000 live births, a figure driven by three main issues: sepsis, eclampsia, and hemorrhage [2]. In 2018, the Zimbabwe Ministry of Health and Child Care (MOHCC) registered 409 maternal death notifications. Postpartum hemorrhage (PPH) was a major direct cause of death among these [3], accounting for 26% of maternal deaths of women of all ages.

Encouraging facility deliveries has been a mainstay of the global strategy to reduce maternal mortality, which Zimbabwe has promoted. While there has been a notable increase in deliveries occurring in health facilities in Zimbabwe, from 65% in 2011 to 85% in 2019 [2], not all facilities are equipped at all times with the staff and supplies to address complications that may arise. On the other hand, a sizable proportion of deliveries still occur in the community, where transport to facilities can be difficult. Some families delay seeking care deliberately due to religious beliefs [4]. OH in particular is an emergency that requires timely intervention, as unabated bleeding quickly can lead to hypovolemic shock, organ failure, and death.

The lens of the “three delays” provides insight by sorting obstacles in access to emergency obstetric care into three categories; 1) time taken in deciding to seek appropriate medical help for an obstetric emergency, 2) time taken in reaching an appropriate obstetric facility, and 3) time taken in receiving adequate care when a facility is reached [5]. In Zimbabwe, the “first delay”, the maternal-level decision to seek care, is estimated to be a strong contributor to about 49% of preventable maternal deaths [3]. The second and third delays however, the amount of time that elapses between seeking care and arriving, and time to receiving definitive treatment, are thought to contribute to the majority of OH deaths.

The Non-Pneumatic Anti-Shock Garment (NASG) is a first aid tool that can halt and reverse hypovolemic shock secondary to OH. NASG is a lightweight compression wrap that is reusable and reasonably simple to apply, making it quite suitable for low-resource settings. While the NASG alone is not a treatment for OH, it buys essential time to transport a woman to a facility that can provide the services she needs, and within facilities that can provide those services, to assure that adequate time is available to prepare and deliver life-saving obstetric emergency care.

Numerous studies have shown the clinical effectiveness of NASG in reducing blood loss, improving recovery time from shock, decreasing mortality, and reducing incidence of extreme adverse outcomes (EAOs) [6–10]. In 2012, The World Health Organization (WHO) evaluated available evidence about the NASG, and recommended it as a temporizing measure while awaiting transfer [10], but uptake of the recommendation has been slow. Some of the evidence of NASG clinical efficacy was generated in Zimbabwe. A randomized controlled trial (RCT) that was conducted in Zimbabwe and Zambia, found 46% reduced odds of mortality and a 54% reduction in composite extreme adverse outcomes (EAO) [10]. This RCT, conducted from 2007 through 2012, was limited to urban and peri-urban settings, and similar to the bulk of studies involving the NASG, did not study the process of incorporating the NASG into the public health system. While many of the large-scale NASG clinical trials similarly have focused on their use at hospital level in urban settings, pilots conducted by The Clinton Health Access Initiative (CHAI) have introduced the NASG at the health center level in Ethiopia and at the community level in Nigeria, with excellent results [11–13].

Once an intervention is shown to have efficacy, cost is one of the next factors given priority for evaluation. Use of the NASG in management of hypovolemic shock secondary to OH has been shown to reduce poor clinical outcomes, but also to be highly cost-effective [14]. “Cost-effective,” however, does not necessarily mean affordable, particularly to a public health system in a lower income country. The price of a single NASG, excluding shipping costs was $68.50/unit in 2013, or $145 from the UNFPA Product Catalog [15]. Though the NASG is reusable, anecdotally, that price was daunting for a tool that needs to be in place in sites throughout a catchment area, and was a noted as deterrent in discussions of potential scale-up of NASG in Zimbabwe. Additionally, concerns over how many uses one garment could withstand added to the impression that NASG was not an affordable solution. In response, CHAI, NASG suppliers, Ministries of Health, and non-governmental organizations collaborated in market shaping endeavors. As a result, the price was reduced to $41.55 per garment in 2014 [16]. Further, the lower-priced garment was a new version, of quality superior to its higher-priced predecessor, made with a more durable material that can withstand up to 144 washes and stretch-and-recovery cycles, as documented in an independent laboratory report provided to the manufacturer [17]. With the reduced unit price and improved durability, the cost per use of NASG is now $0.29 before shipping [11], a 78% reduction in the lowest-available cost per use of the NASG. Taken together, the documented clinical efficacy of the NASG, the substantial price reduction for a higher-quality garment, and savings estimated to be $21.78 per disability-adjusted life-year (DALY) averted, these factors suggest that the NASG is a life-saving, cost-effective tool that could improve the effectiveness of obstetric emergency response if introduced into national programs. However, scale-up plans for Zimbabwe or most other public health systems in low- and middle-income countries (LMIC) have not been initiated [18]. After the end of the NASG RCT in 2012, the government of Zimbabwe did not begin procurement and use of NASG in their health system. Anecdotally, cost, logistics and operational concerns, such as clinical skills and referral systems, were barriers that prevented the Ministry of Health and Child Care from introducing the NASG. This slowdown in progress around NASG is what led the team to embark on this study, with the intention of gathering evidence about whether introduction at primary health care level can be achieved at cost that is affordable and with logistical challenges that are manageable.

In order to better translate the clinical and economic evidence supporting the NASG into practical action in the public health system of a lower income country, the NASG was introduced in a naturalistic setting and the process was documented through a multi-dimensional evaluation, including incremental costs associated with the introduction. Private sector, for-profit facilities were not included in the evaluation because their experiences were not seen as directly relevant to questions about how the public health system in Zimbabwe could fare in introducing the NASG.

## Methods

The Institutional Review Board of The Medical Research Council of Zimbabwe approved this study in December 2018. The approval number is: MRCZ/A/2400. Written informed consent was obtained from participating health workers and oral informed consent was obtained from participating patients. This decision was made in collaboration with the Medical Research Council of Zimbabwe. Two key points considered were: 1) Hypovolemic Shock from obstetric hemorrhage is a medical emergency that requires fast action to save lives. In that context, obtaining written consent seemed like a potential risk to safety of patients; 2) the Medical Research Council of Zimbabwe had prior positive experience with the NASG randomized control trial in Zimbabwe, where verbal consent using a very similar script was obtained from enrolled patients.

This was an observational study to evaluate the process of incorporating the NASG into the obstetric emergency response within the Zimbabwe public health system using an explicit implementation strategy. Study sites were facilities within a discrete geographical area of Zimbabwe, comprising the public health system of Hurungwe Province and its referral hospital in the adjacent district. The study was conducted prior to national introduction of NASG, with the intent of providing operational guidance for future policy and program planning. Data collection was aligned to the RE-AIM framework, looking at the process of the intervention in terms of its reach, effectiveness, adoption, implementation and maintenance [19].

### The RE-AIM framework

Originally presented in 1999, the RE-AIM framework organizes evaluation into five domains, which, examined together, estimate an intervention’s public health impact [20]. While in research settings NASG has been shown to be of great clinical value, its uptake has been minimal, partially due to lack of evidence that it could work in a real-world environment, outside the controlled, well-resourced conditions of a clinical trial. Assessment utilizing the RE-AIM framework can aid the translation from clinical evidence to widespread implementation by virtue of including domains outside of efficacy and cost-effectiveness. The duration of this study was brief; although mortality was recorded, the process evaluation was not designed to capture statistically significant changes in maternal mortality, which is a relatively rare event requiring large populations and longer durations to assess. Additionally, the study duration was not long enough to utilize the framework to estimate public health impact reliably. However, interpretation through the RE-AIM framework does provide an organized, realistic evaluation of a spectrum of aspects of the process of NASG introduction to a rural public health system in a lower-income country. These organized observations can provide valuable guidance for practical action to take the NASG to national scale. The original RE-AIM domains and how they were adapted for use in this study are outlined in Table 1 below.

**Table 1:** The original RE-AIM framework and adaptation for Hurungwe process evaluation.

Dimension	RE-AIM definition	Application in NASG introduction	Definition as for NASG introduction	Outcome measure
**Reach**	Measurement of participation; the absolute number, proportion and representativeness of individuals who participate in a given initiative	Hurungwe District facilities performing obstetric services invited to be trained in NASG and integrate its use in their obstetric emergency response network	Level of penetration the NASG achieved in the study catchment area	Number of facilities in Hurungwe with a provider well-trained in NASG, Cascaded NASG training, number of NASG uses among cases of hypovolemic shock secondary to obstetric hemorrhage.
**Effectiveness**	Consequences of the initiative, which can be understood in terms of biologic indicators, whether positive or negative, and not excluding quality of life and economic outcomes	The effect of NASG in cases of hypovolemic shock secondary to obstetric hemorrhage, stabilization and recovery; qualitative experience of providers using NASG	Clinical outcomes among cases of obstetric hemorrhage; Provider satisfaction with incorporation of NASG	Number of extreme adverse outcomes among cases of obstetric hemorrhage and whether the NASG was utilized in those cases. Qualitative feedback from providers using NASG.
**Adoption**	The absolute number, proportion and representativeness of settings and individuals who are willing to initiate a program	The extent to which NASG has been accepted from introduction within Hurungwe District, both in terms of facility preparation and actual service delivery	The explicit strategy used for integrating the NASG into current practices within a naturalistic setting	Number of facilities within the catchment network that participated in NASG introduction, how many used it, how many used it correctly. Qualitative feedback on adoption from providers.
**Implementation**	The extent to which a program is delivered as intended	Stepwise NASG introduction and stewardship, followed by each participating facility and by the catchment area as a network	Fidelity to newly introduced processes; The practical details of putting the introduction strategy in place and executing it	Training and testing of HCWs on NASG theory and use, cleaning and folding, logistics for replacement, storage and tracking, documentation; and SOPs for emergency obstetric care and referrals
**Maintenance**	The extent to which a program or policy becomes routine and part of the everyday norms and culture of the implementing entity	Routinization and integration of NASG use, logistics, and monitoring into district obstetric emergency response	Continued fidelity to NASG implementation strategy; cost to introduce and maintain NASG within district-level obstetric emergency response.	Use of Obstetric Hemorrhage Case and Transfer Forms; System capacity to incorporate NASG: log books used, replacement logistics systematized; affordability of introduction and maintenance costs

### Setting and study participants

**Fig 1:**
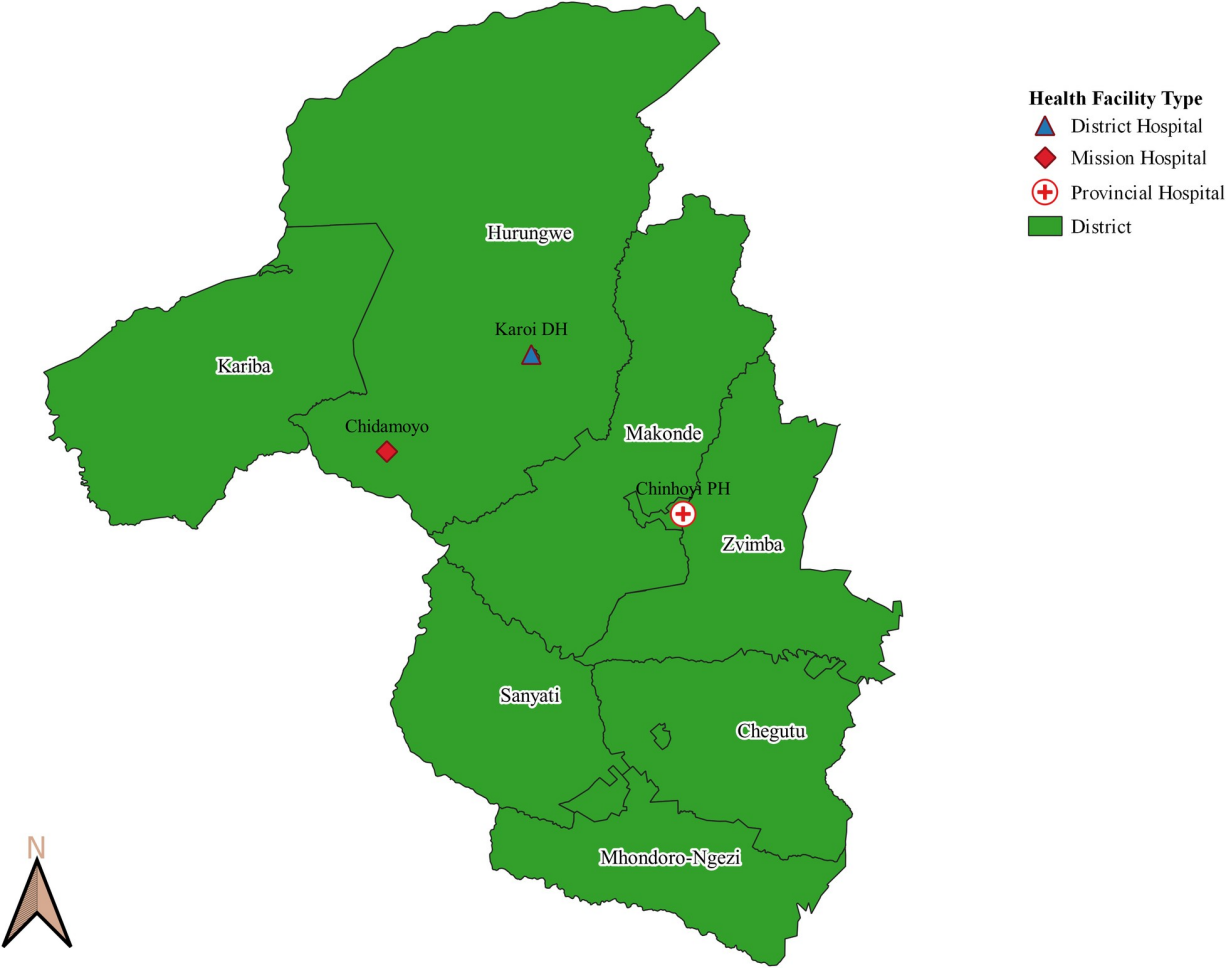
Map of study area with referral facilities marked. Reprinted under a CC BY license, with permission from Brighton Gambinga, original copyright 2020.

In consultation with government stakeholders, Hurungwe District of Mashonaland West Province in north central Zimbabwe, bordering Zambia, was selected for this process evaluation. Reasons for this choice were multi-factorial, including non-urban or peri-urban setting, relative lack of implementation partners, burden of maternal morbidity and mortality, and feasibility of access by road. Hurungwe, with a population of 358,000, is a predominantly rural district in the province; 92% of people reside in rural settings where in general, only primary care is accessed. 13,674 births were reported in Hurungwe district in 2018. Of the 13,476 live births reported, 93.3% were institutional, the largest proportion of which (41.4%) were conducted at primary care facilities; 34.1% of facility births occurred at district and mission hospitals, 11.3% at rural health centers, and 13.1% at rural hospitals. Staff from the provincial health administration, Hurungwe District health management staff and those providing obstetric care at the primary, secondary and tertiary levels participated in the process evaluation. NASG was introduced for use in 34 health facilities: 1 tertiary level, 2 secondary, and 31 primary level facilities. The study area is depicted in the map displayed in Fig 1.

#### NASG introduction into the Hurungwe District obstetric emergency response system

Over the course of three months, NASG was introduced into Hurungwe District through didactic and practical training in a ‘training of trainers’ style, where NASG trainers from the study team trained selected health workers from the participating facilities, and those trainees were expected to cascade NASG knowledge to co-workers at their facilities. The training demonstrated the use of the NASG, the theory behind it, and the necessary steps to introduce it, including how to fill in the study-specific OH case and transfer form, and data and logistics registers. Deployment of NASGs and data and logistics tools was done at the time of the training. For the remainder of the study period, participants were followed up at their facilities. Further, mentorship was delivered through a pre-existing provincial-district level supportive supervisory program. During those visits, NASG skills were observed in health workers through simulated obstetric hemorrhage response, data collection tools were reviewed, mentorship was provided, and informal qualitative feedback from health workers who were trained in NASG. Answers and comments from the health workers were recorded on supervisory visit forms.

Non-medical janitorial staff were also selected to receive training in cleaning and storage of the garments; their role in successful incorporation of the NASG into obstetric emergency response was critical, as the logistics of cleaning, rotating, and tracking garments was paramount to its success in a clinical network. Standard operating procedures for emergency transport and NASG logistics were developed by District staff, supported by the research team. Availability of ambulances, informal arrangements for emergency transport, contact phone numbers and suggested troubleshooting were assessed and mapped in the referral tree of District facilities.

### Data collection

All data collection was paper-based and done continuously throughout the three-month period, from 7 January 2019 to 31 March 2019. To capture as much detail about how the district system responded to OH after NASG introduction, each case of OH presenting to participating facilities was documented, regardless of whether hypovolemic shock developed from the OH, or whether the NASG was used as part of treatment. In addition to clinical details, communication and transportation details were documented: which facility was called at what time, and how long it took for transport to arrive once called.

Other data was collected through NASG logs and clinical registers, and through documentation of supportive supervision that assessed retention and feedback of NASG skills among facility workers. Supportive supervisory visits were conducted by two District clinical supervisors, who were accompanied by two midwives on the study team who are NASG trainers. The pairings were not always the same, and the four debriefed on the findings of each supervisory visit, in order to minimize interobserver variation. In addition to assessing NASG skills, District supervisors also collected informal qualitative data, asking open-ended questions during visits, and recording some comments health workers made about the experience of learning how to use NASG and incorporating it into their pre-existing obstetric emergency response protocols. This einfomral qualitative data was collected from health workers who were trained, regardless of whether or not they had used NASG clinically yet. Clinical registers were augmented by study-specific forms to document clinical details of obstetric hemorrhage cases, such as estimated blood loss, signs of hypovolemic shock, treatments provided, if NASG was applied, if applied when and where removed, and clinical outcomes, such as shock recovery. Incremental costing was done through monitoring of expenses related to NASG introduction, including district sensitization, NASG and sundries procurements, service provider training, as well as post training follow up and supportive supervision visits.

## Results

### Reach

Reach, or penetration of the intervention within the study area, had two key aspects in this study: saturation of NASG skills and units throughout the district, and clinical use of the NASG. Saturation of NASG and skills for its use among the participating facilities was impressive, with 100% of participating facilities demonstrating both basic command of NASG use and cascaded training on the NASG to providers who did not attend the NASG introduction training. This aspect of reach is also represented by distribution of the garments within the district network: 9 garments to the tertiary care referral hospital, 6 each to the two secondary care hospitals, and 3 each to 2 rural hospitals and 29 rural primary health facilities. Numbers of garments at the higher level facilities had to be adjusted up soon after introduction, after it became clear to staff at those facilities that the NASG was as useful internally in both comprehensive and basic emergency obstetric care facilities as for facilities referring patients up, given the high volume of deliveries, inward referrals, and utility of the NASG to stabilize a woman while preparing for definitive treatment made.

Reach was also assessed through the lens of NASG availability, accessibility and equity. Availability of the NASG in the selected facilities ensured that the intervention was an option for the women who may have needed it during the study period. Access to the intervention was assured by the availability of service providers skilled in use of the NASG in all the participating facilities. One detractor to this aspect of reach was that, due to scheduling conflicts, the intervention commenced two weeks later at the tertiary facility than at the rest of the participating facilities, which was consequential in terms of OH response at that facility during the lag period. Equity in access to the NASG was achieved by the high level of reach of the NASG. To increase equity and offset the unpredictable nature of OH, the NASG was availed to all participating facilities, regardless of volume of births occurring at the facility, so that the intervention was available for all women who needed it, regardless of the social context or geographic location of the woman or facility within the catchment area.

**Fig 2:**
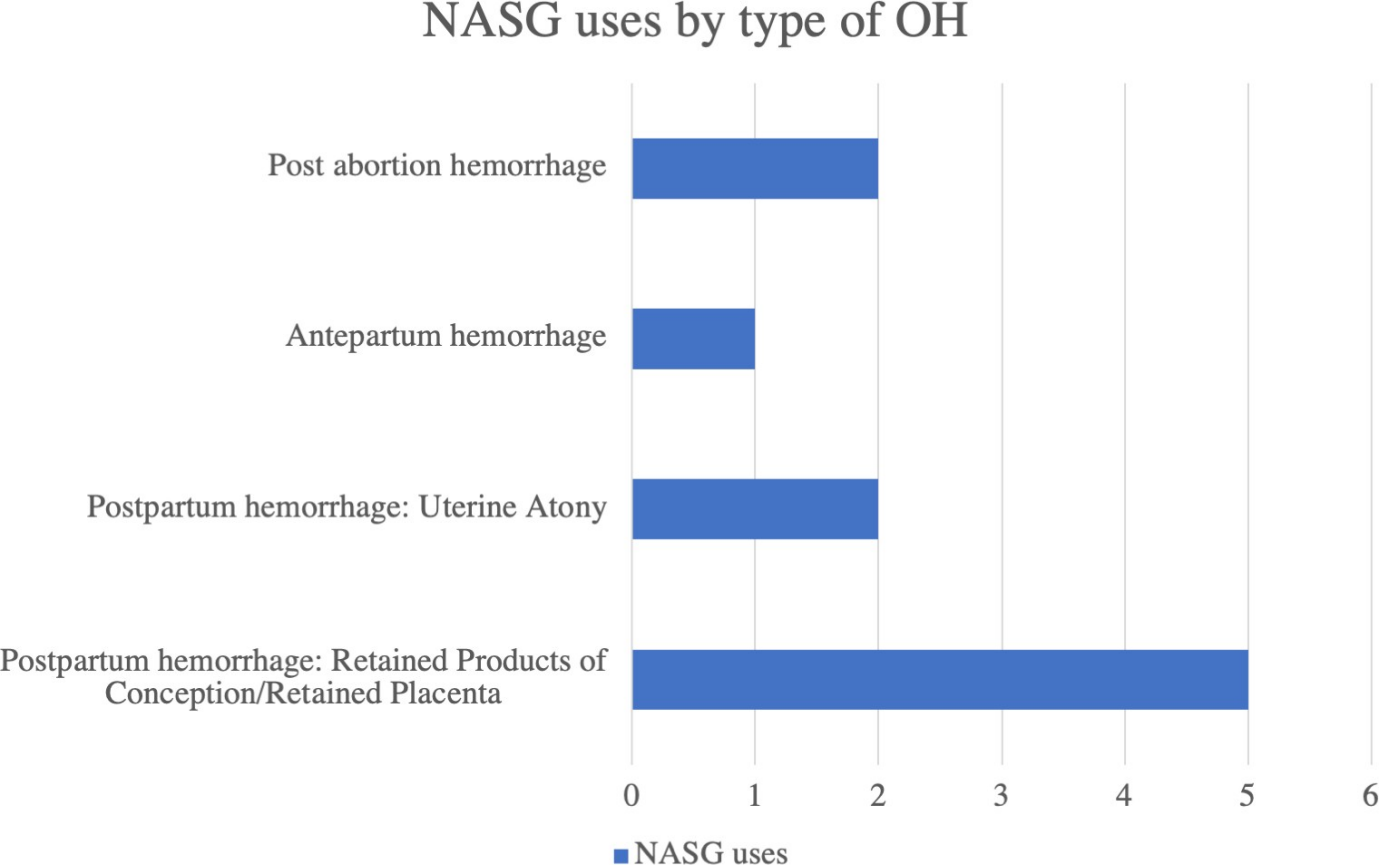
Number of NASG uses by cause of obstetric hemorrhage.

When examining reach of the NASG as evidenced by appropriate clinical application, the results were promising but less universal. Among the 11 cases of hypovolemic shock that were reported during the study, the NASG was applied in 10 cases. The case where NASG was not used, despite documented hypovolemic shock secondary to OH, occurred at a referral level facility. Nonuse of the NASG at that facility may have resulted from the impression that the NASG could only be used for stabilization in preparation for and during transport, applied at facilities where the needed definitive treatment was not available. This scenario for use was emphasized during NASG training, to highlight that the NASG is a first aid device useful while transferring patients from lower to higher levels of care. The case of nonuse suggests a need to clarify the utility of applying the NASG as a clinical management tool in any facility, regardless of patient transfer, to gain time to prepare for and deliver definitive treatment, facilitating a quick recovery from shock. The types of OH that led to the NASG uses, shown in Fig 2, were predominantly types of PPH as expected, however, its use in cases of antepartum and post abortion hemorrhage evidences clear understanding of the scope of NASG indications on the part of the newly trained providers. The majority to NASG uses, 7 of 10, were initiated with transfer to a higher-level facility for definitive care after stabilization as part of the care plan.

One finding of interest related to the clinical use aspect of reach related to the observed incidence of OH. While the true rate of OH in Hurungwe District is unknown, estimation of the anticipated number of OH cases was made using reported rates from the global literature alongside 2018 Hurungwe statistics. This led to an estimation that 69 cases of OH were anticipated to occur during the study period, with approximately 15% progressing to hypovolemic shock. During the study, 78 cases of OH did occur, 113% of the anticipated total, with 14, 17.9% of them resulting in documented hypovolemic shock. A caveat to note is that there were 3 OH cases that were not documented as hypovolemic shock, but were flagged by clinical reviewers as potentially undiagnosed shock cases. If those 3 cases were indeed hypovolemic shock, the rate would have been 21.7%. These figures contribute to and help validate current thinking about estimation of OH in resource-constrained settings.

The 3 cases of potentially undiagnosed hypovolemic shock mentioned above received definitive treatment at the facilities without the benefit of NASG application, and all three women recovered. These potential NASG cases received surgical care to arrest hemorrhage immediately without the NASG and were referred or managed post operatively without the NASG. This finding indicates the need to strengthen identification of early signs of hypovolemic shock. Since hypovolemic shock was not diagnosed in those three cases of potentially missed opportunities for NASG, not using the NASG in those cases technically was appropriate. While staff at rural facilities did use NASG for stabilization during transfer, the district hospital did not use NASG in one case where it would have been appropriate while preparing for definitive care within the hospital, indicating the need to reinforce use of the NASG within a facility at referral level.

In two of the NASG cases, women received definitive treatment, but the management was varied, providing insight into the contextual importance of NASG. In one case, the NASG was applied and treatment given, but there was also need for blood transfusion. Due to lack of availability of blood, the woman was managed with hematinic medication in the intensive care unit while wrapped in a NASG, until she became stable. In a similar case at secondary level, stabilizing treatment was provided, and the patient was referred to the next level for blood transfusion after removal of the NASG.

### Effectiveness

Effectiveness, as defined for the NASG introduction to Hurungwe District, is measured by the number of maternal deaths or extreme adverse outcomes (EAO), including emergency hysterectomy, and organ failure. Based on 2018 data from Hurungwe District, 3–4 EAO secondary to OH and 2–3 maternal deaths from OH were anticipated. Among the 10 of 11 hypovolemic shock cases where a NASG was utilized, all were cases in which NASG was used appropriately, resulting in zero deaths or EAO. With correct clinical use, zero mortality and zero other EAO, effectiveness as defined in the RE-AIM framework for this process evaluation was high.

**Fig 3:**
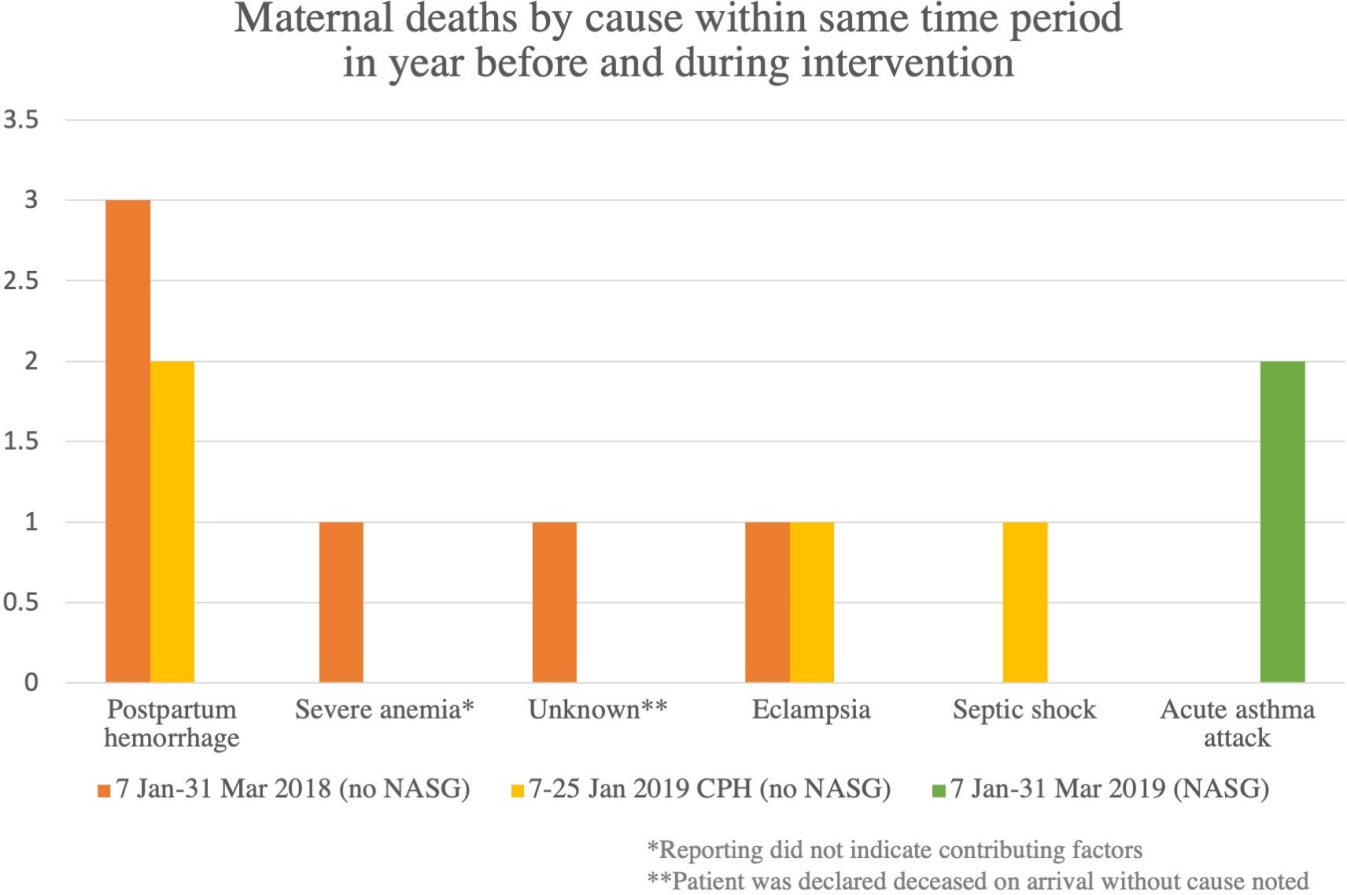
Maternal deaths by cause in time periods before and after NASG introduction.

The estimate of expected all-cause maternal death during the study period, was 4–5. During the same time period in the year prior to the study, six maternal deaths, three of which were from PPH were reported in Hurungwe; during the study period after NASG was introduced, there were no deaths from OH. This analysis excludes maternal deaths that occurred at the tertiary hospital during the two-week period in January 2019 when NASG had been introduced to participating Hurungwe facilities, but not yet introduced to the tertiary facility. Two of the maternal deaths from the tertiary hospital that occurred in that time period were attributed to PPH, and therefore may have been averted if introduction of the NASG had been done at the same time for all participating facilities. After NASG was introduced in all participating facilities, there were two maternal deaths before the end of the study period, both from indirect causes. Fig 3 shows the number of deaths by cause in three categories: first, all maternal deaths in Hurungwe in the first quarter of 2018, second, maternal deaths that occurred at the tertiary care facility in the two-week lag time between introduction of NASG to district facilities and to the tertiary facility, and third, maternal deaths in the first quarter of 2019 excluding those from the tertiary facility in the two weeks lag period. Overall, there were fewer maternal deaths in the study period after full NASG introduction than there were in the same time period the year before.

Perspectives of participating health workers were also considered as a reflection of effectiveness, because without acceptance of the new tool it will not be used, in which case it could not be considered effective. Overwhelmingly, the comments by health workers made and documented during supervisory visits were positive, and reflected appreciation of how practical the NASG can be. Not all feedback was without concern, however. One health worker expressed doubt that everyone would remember the correct way to apply the NASG, mentioning that it would be difficult for everyone to apply it, “without forgetting to do a snap test.” Another nurse was concerned about, “lack of knowledge to those not trained, especially at a hospital with a big number of staff. Also, it is a challenge to apply the NASG when you are alone.”

Positive feedback was more common. One nurse from a primary level rural facility pointed out that the NASG doesn’t interact with drugs, and “has no after effects as long as it is used on the right patient at the right time.” Another expressed commitment to exploit the stabilizing effects of the NASG while preparing treatment: “NASG is a first priority before giving all other required medicine, soon after quick check and observations; it’s one of the major devices for preventing shock.” Another nurse recognized the potential impact of expanding availability of NASG: “My hope is for NASGs to start from the village to prevent maternal death.”

### Adoption

All levels of care used the garment at least once during the study, however, due to the limited timeframe, not all of the 34 facilities demonstrated clinical use of the garment. However, through mentorship visits, all facilities demonstrated NASG integration into OH response planning. Among the 10 NASG uses that occurred during the study period, the garment was used correctly in each case.

Among providers, the commitment to incorporating the NASG, regardless of whether it had been used in obstetric emergency response yet, was evident. One primary care nurse from a rural health center that did use the NASG commented that, “the NASG can be applied by anyone as long as they are trained on how to do it correctly. OH is not easy to predict. NASGs being applied early leads to the best outcomes.”

### Implementation

High fidelity to implementation plans was observed overall, especially in terms of training workers from all cadres involved in a range of service delivery, including anesthesiologists, drivers and cleaning staff. The most challenging points in implementation were related to operational issues: access to emergency transport for women in shock and logistics for NASG replacement. The approach to introduction of the NASG involved allowing the health workers themselves to create the networking procedures such as how to secure transport, who to call for referrals and garment replacement. For example, the NASG rotation and replacement system was established by the District administrative and health worker team, making plans that they felt would work best in their practice environment. The standard procedure as planned was for the replacement NASG to be sent in the ambulance that collected the patient from the basic emergency obstetric and newborn care (BEmONC) facility, through communication between the nurse managers of the Maternity Wards. The practical experience proved that these procedures were difficult to adhere to, and required ad hoc adjustments. Qualitative feedback during supervisory visits highlighted transport access issues related to shortages of vehicles and fuel availability, but rather than referring to those factors as implementation challenges, providers referred to those chronic challenges as motivational factors to incorporate use of the NASG to buy time to execute a safe emergency transport. One health worker talked about how incorporating NASG allows less skilled workers to assist in immediate response to a patient who has gone into hypovolemic shock from obstetric hemorrhage: “it can be used by any other staff available at the facility who are trained to use it, as compared to drug administration which can only be done by a nurse or doctor.” Another primary level provider alluded to utility of NASG while arranging referrals, remarking: “A NASG is an effective device at grass root level that can be used while awaiting further management of patient to a higher level of care.” None of the implementation challenges were out of the ordinary for the introduction of a new tool that required a coordinated network of providers.

### Maintenance

Despite the brevity of the study period, important signals related to maintenance of the NASG were clear. For example, use of the NASG was formally integrated into the District PPH management package, both in policy and in practice. One NASG was dedicated to the PPH management emergency kits at all facilities. Similarly, NASG was integrated into the antenatal health education sessions as a topic, promoting acceptance and longevity of NASG use by familiarizing all users of maternal services in Hurungwe District with its appearance and purpose.

NASG logbooks remained available and filled in at facilities. The standard referral forms were easily annotated to include NASG information in lieu of the separate OH case and transfer form created for use in the process evaluation. Most facilities conducted in-service training on the NASG for their co-workers, adding 127 trained workers to the 119 trained through the introduction sessions, more than doubling the NASG-skilled workforce through cascade learning.

Through pre-existing district mentorship and supportive supervision, OH response drills that include NASG application became regular practice sessions, both reinforcing skills, teamwork, and extending capacity. Additionally, a mission hospital, one of the secondary care facilities and the only non-government facility in the study, took the initiative to plan for training and equipping a remote clinic that refers in to their hospital with NASG training and garments. This is a clinic which is outside of Hurungwe District, but from which the hospital receives most of their OH clients. The long distance and poor roads between these two facilities resulted in six-hour referral times, thus the need for the NASG was critical and was proactively addressed by this participating facility.

The maintenance costs were also encouraging. The price of the garments used in the introduction was far lower than in previous years, while the quality of garment is greatly improved, allowing for 144 washes without loss of compression. The budgetary needs for replacing garments in the district were therefore quite low [20]. Cost of cleaning solution, vessels and brushes for decontamination and washing were tracked and proved modest at approximately $45 per secondary level site, for supplies that are projected to last for two years. The cascaded model of training, reinforced through pre-existing supportive supervisory visits and regular team drills represented a cost savings as compared to a classroom training model of skills building repeated for different batches of health workers.

## Discussion

Demonstration and evaluation of the process the national health system of a low-income country would need to undertake in order to avail the proven clinical and economic benefits of the NASG has been missing in the global literature. Evidence from controlled study environments, taking place in largely urban areas, has not been intended to provide insight into introduction of NASG in real-world low- and middle-income public health systems settings, rather, these efforts were more focused on the previous step, showing that NASG is efficacious and valuable clinically and is cost-effective [7–10]. Documentation of the Hurungwe District experience of integrating NASG into its pre-existing obstetric emergency response uncovered important points of congestion in development of a sustainable system using the NASG, and some targets for general quality improvement, such as increased recognition of early signs of hypovolemic shock. The process evaluation provided an opportunity to document the actual rate of obstetric hemorrhage in Hurungwe, and an important demonstration of clinical benefit of NASG use in a naturalistic environment. Incorporation of NASG use into OH response was done with apparent ease by health workers at all three levels of care included in the study. However, the supportive supervision visits uncovered several areas that would need more attention in subsequent introduction and scale up of NASG use, including reinforcement of intra-facility use case for NASG, routine team obstetric emergency response drills, identification of signs of shock, and more consistent and thorough documentation for both patient hand-over as well as logistics for NASG washing and replacement.

Because use of NASG mostly involved referral of a patient to another facility, the need to replace used NASGs at the facility where it was applied brought to light the common difficulty of providers from different facilities being able to exchange material goods. While the ideal protocol, as laid out by the participating providers, was to have a clean NASG on the ambulance to give to the referring facility when the patient in a NASG was picked up, the reality was that ambulances and fuel were in short supply. Often relatives secured private transport in lieu of continuing to wait hours for the ambulance to arrive, or ambulances may come from another call, without the opportunity to pick up the replacement NASG from the referral facility first. It is also possible that being early on the learning curve of NASG incorporation, practical solutions, such as having a clean NASG aboard every ambulance, did not have time to gain traction. Flow of the NASG between Departments in the tertiary care Provincial Hospital was another difficulty noted. It became clear that wider sensitization, among many wards within the hospital, was needed to ensure that garments did not become lost. We did not observe any NASG replacement difficulty related to a sense of ownership of garments; each NASG was labeled with a provincial ID number, which was used in the NASG logbooks to indicate intake and outflow of each individual garment.

Another important lessoned learned was that as much as NASG proved very useful in buying time to wait for emergency transport that was difficult to obtain in the study area, there were at least two NASG uses where life-saving time was provided by the NASG while access to definitive treatment, in these cases, blood transfusion, was secured. Non-application of the NASG in preparation for surgical or medical intervention at the tertiary facility as well as varied use of the NASG as a management tool in cases where the definitive treatment was provided in phases, suggested a gap in the appropriate use of the NASG. While the application of NASG to patient in need of treatment only available elsewhere was consistent, only one facility demonstrated use of NASG when definitive treatment was available on site, and referral of the patient was not expected. Further, it is notable that in no case was the NASG removed before definitive treatment for the OH was provided and the patient was stable as per NASG removal clinical guidance.

The incremental costs associated with NASG introduction were modest and affordable with respect to the District health budget. However, it was noted that the cost of introduction of the NASG was interrelated with the health system’s strength in the sense that where basic commodities for obstetric service delivery, such as blood pressure cuffs or cleaning supplies were lacking, the cost of introduction increased.

The way health workers embraced integration of the NASG into their emergency response standard operating procedures, both in preparation and practice, indicated a clear understanding of the theory behind NASG use, and increased the likelihood that the intervention would become normalized in obstetric practice. The improved clinical outcomes observed during the study period further confirmed this assertion. No resistance to use of the NASG from managers or frontline health workers was apparent.

### Limitations

This process evaluation had several limitations to note. Due to the brief time period of the study, the public health impact score of NASG introduction could not be calculated as proposed in the original RE-AIM evaluation model. Duration of 6 to 12 months is recommended before calculating public health impact score, which is the product of the five RE-AIM dimensions [21]. The duration of the study itself was a limitation, in that change in the dimension over time was not possible to observe. While the clinical effectiveness of NASG in this process evaluation was effectively 100%, the time period of the study was too short to assess impact on maternal mortality.

## Conclusion

In the first process evaluation of NASG introduction to a natural public health setting in rural Zimbabwe, it was found that in the domains of reach, effectiveness, adoption, implementation, and maintenance, NASG introduction dovetailed very well with pre-existing systems for obstetric emergency response, and improved clinical outcomes for women who suffered from hypovolemic shock secondary to OH. Uptake of NASG was nearly universal and feedback from trained health workers was positive. Further, the cost of introduction was affordable, despite the need for extra expenditure on basic commodities used in delivery of all obstetric care. Scale-up of the NASG in the Zimbabwe public health system can be undertaken with careful attention to buttressing systems such as mentorship, drills, documentation and logistics.

## Supporting information

S1 Fig (JPG)

S1 Data (XLSX)
