# Supplementary figures and images for: Applying the RE-AIM framework in a process evaluation of the introduction of the Non-Pneumatic Anti-Shock Garment in a rural district of Zimbabwe
Source: PLoS One. 2021 May 20;16(5):e0251908. doi: 10.1371/journal.pone.0251908 (PMC8136624; doi:10.1371/journal.pone.0251908)

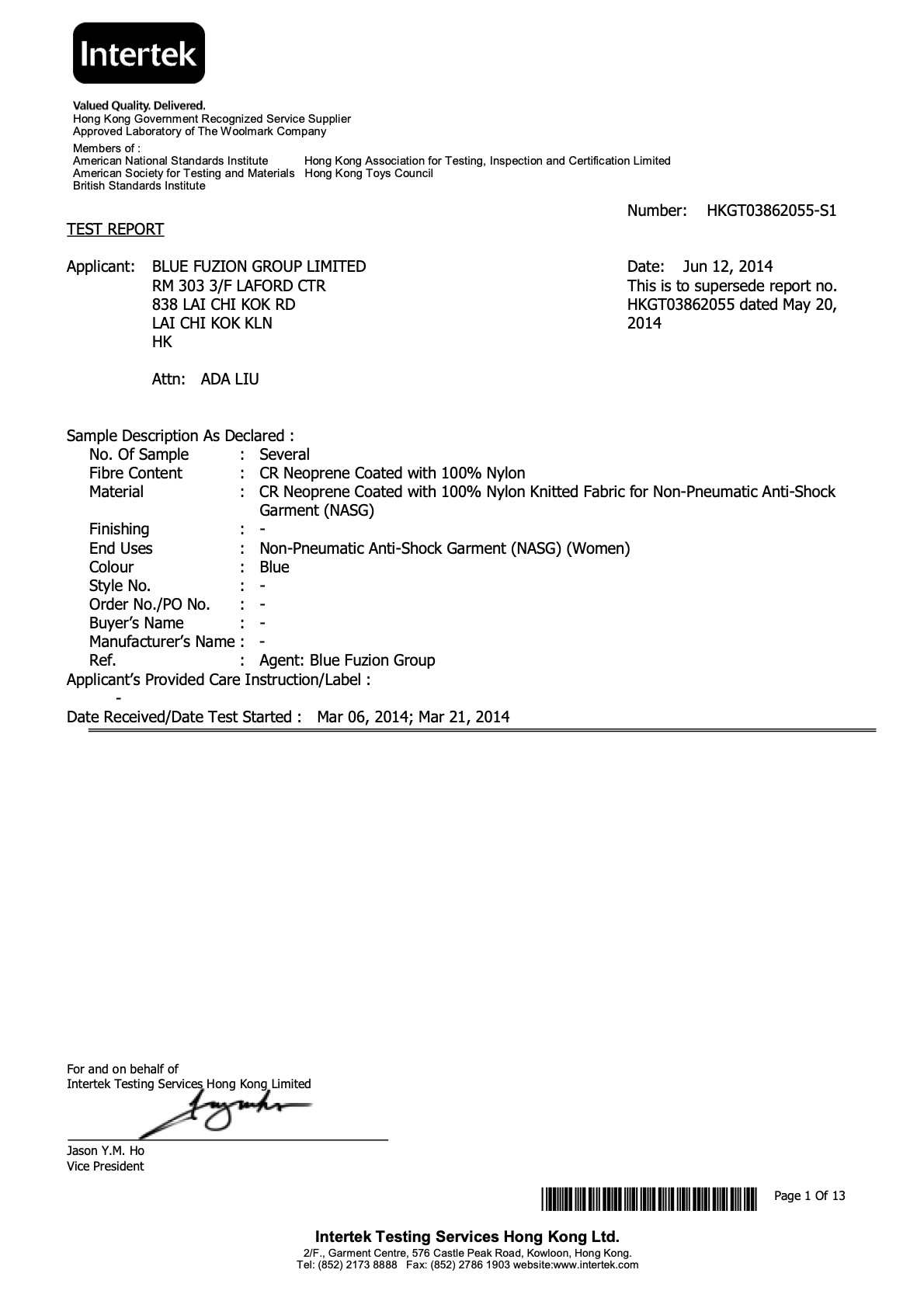

Supplement: S1 Fig — (JPG) [file pone.0251908.s001.jpg]
